# Chromatin as a sensor of metabolic changes during early development

**DOI:** 10.3389/fcell.2022.1014498

**Published:** 2022-10-10

**Authors:** David Pladevall-Morera, Jan J. Zylicz

**Affiliations:** Novo Nordisk Foundation Center for Stem Cell Medicine, reNEW, University of Copenhagen, Copenhagen, Denmark

**Keywords:** metabolism, epigenetics, chromatin, cell fate, epi-metabolomics, early embryonic development, nuclear metabolism

## Abstract

Cellular metabolism is a complex network of biochemical reactions fueling development with energy and biomass; however, it can also shape the cellular epigenome. Indeed, some intermediates of metabolic reactions exert a non-canonical function by acting as co-factors, substrates or inhibitors of chromatin modifying enzymes. Therefore, fluctuating availability of such molecules has the potential to regulate the epigenetic landscape. Thanks to this functional coupling, chromatin can act as a sensor of metabolic changes and thus impact cell fate. Growing evidence suggest that both metabolic and epigenetic reprogramming are crucial for ensuring a successful embryo development from the zygote until gastrulation. In this review, we provide an overview of the complex relationship between metabolism and epigenetics in regulating the early stages of mammalian embryo development. We report on recent breakthroughs in uncovering the non-canonical functions of metabolism especially when re-localized to the nucleus. In addition, we identify the challenges and outline future perspectives to advance the novel field of epi-metabolomics especially in the context of early development.

## 1 Introduction

**FIGURE 1:**
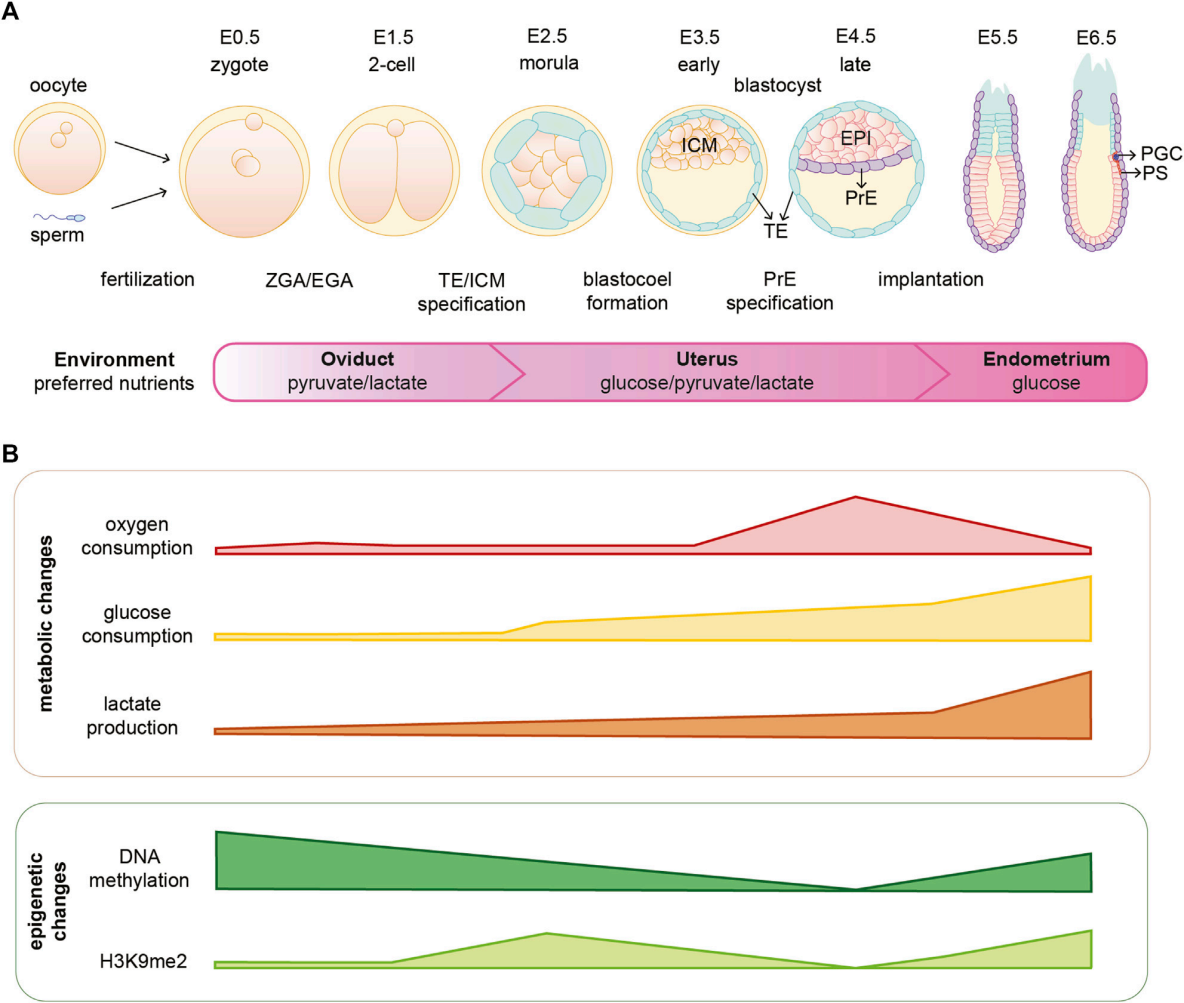
Mouse embryonic development is coordinated with metabolic and epigenetic changes. **(A)** Schematic illustration of early mouse embryogenesis from fertilization until implantation (E0.5-E6.5) and crucial developmental events. The embryo moves through different environments within the female reproductive system and several metabolites are essential for embryo development in different stages. **(B)** Oxygen availability and consumption vary depending on the stage of embryonic development. Furthermore, the embryo undergoes metabolic and epigenetic reprogramming during the first days of development. EPI, epiblast; H3K9me2, dimethylation of histone H3 lysine 9; ICM, inner cell mass; PGC, primordial germ cells; PrE, primitive endoderm; PS, primitive streak; TE, trophectoderm; ZGA/EGA, zygotic genome activation/embryonic genome activation.

Fertilization is the moment when oocyte and sperm merge to form a zygote, which will undergo a series of divisions giving rise to the different cell lineages and eventually the adult organism. Early embryonic development is accompanied by rapid and dynamic epigenetic changes that regulate the expression of developmental genes and prime cells for lineage commitment, as summarized in Figure 1. Formation of the zygote is followed by a global DNA demethylation that occurs up to the blastocyst stage. Subsequently upon implantation there is a wave of *de novo* methylation of the DNA (Borgel et al., 2010). The fundamental function of such epigenetic rewiring in regulating and coordinating embryo development is continuously being updated and exciting new roles are being described in that context (Messerschmidt et al., 2014; O’Neill, 2015; Gökbuget and Blelloch, 2019). However, how these processes are initiated and coordinated is to-date not fully understood. Interestingly, studies in the past years suggest that metabolism might be playing an important and active role in orchestrating early embryonic development. Not only by the fact that metabolism provides the embryo with energy and biomass to sustain fast proliferation, but also by directly shaping the epigenetic landscape of the early embryo and, therefore, linking metabolism with gene transcription.

**FIGURE 2:**
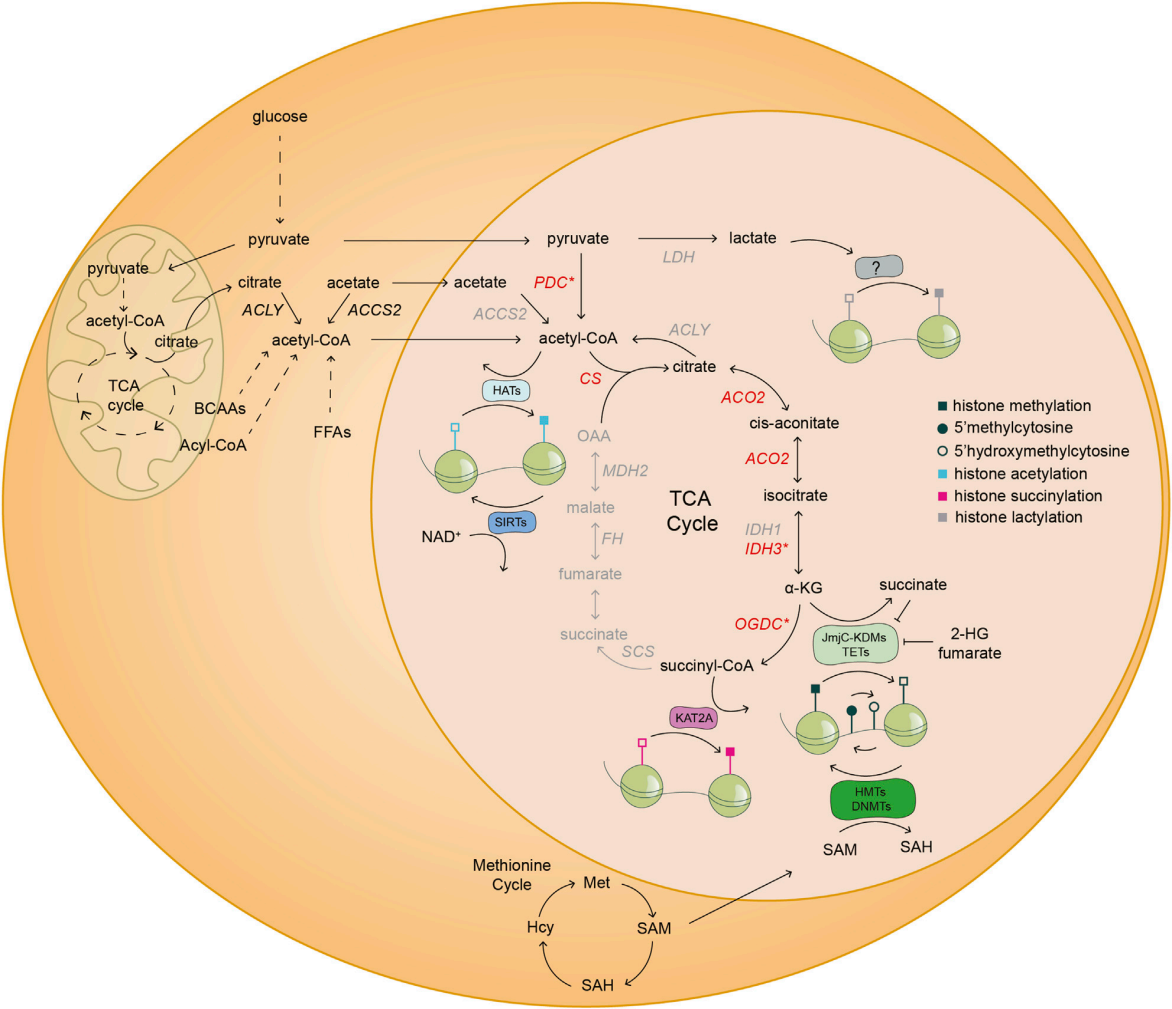
Nuclear metabolism shapes the cellular chromatin landscape. Schematic illustration of the coupling of nuclear metabolism with epigenetics. The existence of a nuclear metabolic sub-network of the TCA cycle (black) and nuclear localization of distinct metabolic enzymes (red) have been reported in mouse embryos and/or mESCs. The asterisk (*) denotes that at least one of the subunits of the highlighted enzymatic complexes are found inside the nucleus of mouse embryos and/or mESCs. Specifically, PDH in the case of PDC; IDH3G from the IDH3 complex; and OGDH subunit from the OGDC. The other half of the TCA cycle and several other metabolic enzymes (grey) have only been described in the nucleus of cancer cells. The TCA cycle metabolite α-KG is a co-factor and substrate of JmjC-KDMs and TETs, which are histone lysine demethylases and DNA demethylases, respectively. Conversely, other TCA cycle intermediates, such as fumarate and succinate, and the metabolite 2-HG, are inhibitors of JmjC-KDMs and TETs. SAM derived from one carbon metabolism is a cofactor of HMTs and DNMTs, which add methyl groups to histone lysine residues and DNA, respectively. The complex OGDC catalyzes the conversion of α-KG to succinyl-CoA, which is used by KAT2A to succinylate specific histone residues in cancer cells. Acetyl-CoA derived from various metabolic reactions is used by HATs to transfer acetyl groups to histones. On the contrary, SIRTs consume NAD^+^ to deacetylate histone residues. Finally, lactate derived from anaerobic glycolysis might be used by yet undescribed epigenetic modifying enzymes, for histone lactylation. ACCS2, acetyl-CoA synthetase 2; ACLY, ATP-citrate lyase; ACO2, aconitase 2; α-KG, alpha-ketoglutarate; BCAAs, branched-chain amino acids; CS, citrate synthase; DNMTs, DNA methyltransferases; FFAs, free fatty acids; FH, fumarate hydratase; HATs, histone acetyltransferases; Hcy, homocysteine; IDH1, isocitrate dehydrogenase 1; IDH3G, isocitrate dehydrogenase 3 subunit G; JmjC-KDMs, Jumonji C-domain lysine demethylases; KAT2A, lysine acetyltransferase 2A; KMTs, lysine methyltransferases; LDH, lactate dehydrogenase; MDH2, malate dehydrogenase 2; Met, methionine; NAD^+^, nicotinamide adenine dinucleotide; OAA, oxaloacetate; OGDC, oxoglutarate dehydrogenase complex; PDC, pyruvate dehydrogenase complex; SAH, S-adenosylhomocysteine; SAM, S-adenosyl-L-methionine; SCS, succinyl CoA ligase; SIRTs, sirtuins; TETs, ten-eleven translocation methylcytosine dioxygenases; 2-HG, 2-hydroxyglutarate.

The interplay of metabolism and epigenetics is achieved by intermediates of metabolic reactions that can act as substrates, co-factors or inhibitors regulating the activity of chromatin modifying enzymes (see Figure 2) (Katada et al., 2012; Lu and Thompson, 2012). We refer to these molecules as epi-metabolites. For instance, *in vitro* alpha-ketoglutarate (αKG), an epi-metabolite and intermediate of the tricarboxylic acid (TCA) cycle, regulates the pre-implantation naïve state of mouse embryonic stem cells (mESCs) (Carey et al., 2015; Tischler et al., 2019); while *in vivo* αKG is vital for zygotic genome activation (ZGA) of the mouse embryo (Nagaraj et al., 2017). Therefore, evidence is gathering that the metabolic state of cells and the embryo should be taken into account as another layer of regulation of early embryonic development. In this review, we describe the metabolic differences between distinct embryonic tissues and pre-implantation stages. In addition, we discuss the most recent advances in epi-metabolomics during early embryonic development and the existence of a nuclear metabolic sub-network that might have implications in regulating cell states. Together, recent advances substantiate the hypothesis that chromatin acts as a sensor of cellular metabolic states thus allowing for multi-level coordination of developmental processes.

## 2 The role of epigenetics in regulating early embryonic development

Mouse and human embryonic development start with the fertilization of the egg by the sperm and formation of the zygote (1-cell stage). Subsequently, the zygote undergoes several rounds of divisions and proceeds through the morula, blastocyst, gastrula, neurula and finally initiates organogenesis. The early embryonic developmental program between zygote to blastocyst is not entirely conserved between mice and human, since they show differences in gene expression, timing and lineage specification mechanisms (Rossant and Tam, 2017). Towards the end of the 2-cell stage in mice and around the 4-cell stage in humans, the embryo becomes transcriptionally activated in a process known as ZGA or embryonic genome activation (EGA) (Braude et al., 1988; Tesařék et al., 1988; Aoki et al., 1997; Vassena et al., 2011; Asami et al., 2022). The first embryonic lineage commitment occurs at the morula stage after embryos’ compaction, which leads to the formation of two distinct blastomere populations: the trophectoderm (TE) and the inner cell mass (ICM). The second cell fate decision occurs after the blastocyst cavitation. Blastomeres from the ICM are sorted into two populations: the primitive endoderm (PrE) in mice or hypoblast in humans, which is an extraembryonic tissue lying in contact with the blastocoel; and the epiblast (EPI) that is enclosed between the TE and the PrE and will give rise to the embryo proper. Around E7.0 for humans and E5.0 in mice, the three lineages (TE, EPI and hypoblast/PrE) of the blastocyst are defined and the embryo is ready to implant in the uterine wall (Figure 1A) (Braude et al., 1988; Rossant and Tam, 2017). Signaling events regulating early development have been extensively studied over the last decades and have been thoroughly reviewed elsewhere (Perrimon et al., 2012; Menchero et al., 2017; Sonnen and Janda, 2021). This deep knowledge of the signaling milieu allowed researchers to recapitulate many stages of mouse and human embryonic development *in vitro*. By isolating cells from the mouse blastocyst, naïve pluripotent stem cells (mESC), trophoblast stem cells (mTSC) and extraembryonic primitive endoderm stem cells (nEnd) mimicking the ICM, the TE and the PrE respectively, can be obtained (Tanaka et al., 1998; Ying et al., 2008; Anderson et al., 2017; Ohinata et al., 2022). Culture of naïve mESCs *in vitro* is achieved by the addition of GSK3β and MAPK/ERK inhibitors (2i) and leukemia inhibitory factor (LIF) in the so-called serum-free 2i/L media (Ying et al., 2008). A more metastable mESC phenotype was also captured in a serum-based media containing LIF (S/L), where populations dynamically interchange between multiple states (Martin Gonzalez et al., 2016; Morgani et al., 2017). Primed EPI stem cells (mEpiSC) derived from the post-implantation mouse embryo (E.5.0-E8.0) have been established and represent the late post-implantation, peri-gastrulation mouse EPI or primitive streak (Brons et al., 2007; Tesar et al., 2007; Kojima et al., 2014). Additionally, an intermediate state between mESC and mEpiSC known as mouse EPI-like cells (mEpiLC) recapitulating the early post-implantation EPI, can be generated *in vitro* from mESC (Hayashi et al., 2011). Similarly, human stem cell models reminiscent of pre-implantation human EPI with naïve and primed features are also available for research (Thomson et al., 1998; Takashima et al., 2014; Theunissen et al., 2014). To this date, few mechanistic studies have been performed in human embryos, therefore this review will mainly focus on findings from the mouse as a model organism.

Rapid and dynamic epigenetic changes coordinate early embryonic development; however, how these changes are initiated is not fully understood (Borgel et al., 2010; Zylicz et al., 2015; Zheng et al., 2016; Zylicz and Heard, 2020). One of the major waves of epigenetic reprogramming takes place around the time of embryo implantation, when the naïve EPI cells become primed for gastrulation. During this transition, genome-wide *de novo* DNA methylation occurs for the first time after embryo fertilization (Okano et al., 1999; Borgel et al., 2010). In addition, there is a global gain of the repressive H3K9me2 mark, a general decrease of histone acetylation and the establishment of bivalent H3K27me3 and H3K4me3 at developmental gene promoters (see Figure 1B) (Peters et al., 2003; Rice et al., 2003; Lee et al., 2004; Bernstein et al., 2006; Mikkelsen et al., 2007; Efroni et al., 2008; Voigt et al., 2013; Harikumar and Meshorer, 2015). These processes, together with the action of specific chromatin remodeling factors, set the grounds for a predominantly closed chromatin landscape that leads to the establishment of primed pluripotency [reviewed in (Gökbuget and Blelloch, 2019)]. The possible function of epigenetic marks during early embryonic development has been reviewed elsewhere (Hackett et al., 2012; Gopinathan and Diekwisch, 2022).

Epigenetic regulation is crucial to ensure a successful embryonic development. Accordingly, loss of many DNA and chromatin modifying enzymes leads to embryonic lethality before or around the time of gastrulation. For instance, mouse embryos that lack the expression of both *de novo* DNA methyltransferases DNMT3A and DNMT3B show morphogenesis defects due to lack of somites and die shortly after gastrulation (Okano et al., 1999). Additionally, *G9a* ^−/−^ embryos, missing expression of the main mammalian histone methyltransferase mediating the dimethylation of lysine 9 of histone H3, die around E9.5 of development (Tachibana et al., 2005, 2002). Mice lacking EZH2, which is a subunit of the Polycomb Repressive Complex 2 (PRC2) that deposits H3K27me3, die due to gastrulation defects at around 6.5 (O’Carroll et al., 2001; Pasini et al., 2004; Boyer et al., 2006; Shen et al., 2008; Huang et al., 2014). Finally, mouse embryos harboring the loss of *Setdb1/Eset* resulted in embryonic lethality at the peri-implantation stage due to defective lineage specification, resulting in loss of the EPI but expansion of extraembryonic tissues (Dodge et al., 2004). Together, this data points to a vital role of epigenetic regulation at the moment of implantation when cells prepare for cell fate decisions. These findings have been elegantly reviewed elsewhere (Nottke et al., 2009; Shen et al., 2017; Gökbuget and Blelloch, 2019; Jambhekar et al., 2019). However, how precisely is the activity of these chromatin-modifying enzymes regulated? Does the metabolic state of the cells and the embryos’ environment influence their activity? Is chromatin sensing metabolic changes? The following sections of this review describe the metabolic state of the embryo during the distinct pre-implantation and implantation stages. In addition, we focus on how metabolism influences chromatin states and cell fate during early embryogenesis.

## 3 Metabolism of stem cells and early mammalian embryos

To this day, metabolism has been mainly studied in the context of bioenergetics and biomass generation to sustain growth and proliferation of the embryonic tissue. Recent studies shed light on the often-neglected role of metabolism in directly regulating early embryonic development. However, most of the research performed to-date are observations based on the *in vitro* counterparts of different embryonic development stages that might or might not reflect what is happening *in vivo*. Optimizing metabolomics techniques and metabolic flux analysis is crucial to understand the complexity behind metabolic regulation of early embryogenesis and to dissect metabolism with temporal and spatial resolution. This section provides an overview of the metabolic state of the embryo from zygote until implantation. We also discuss the role of hypoxia in metabolism regulation and we highlight similarities between onco- and stem cell-metabolism. Finally, we describe the impact of signaling pathways that are essential for embryonic development, in metabolic regulation.

### 3.1 Defining the metabolic state of the early embryo

Energy in the embryo is mainly obtained through glycolysis or through the activity of the TCA cycle and oxidative phosphorylation (OxPhos) from glucose, pyruvate or glutamine. The choice of the metabolic pathway used for energy production depends on several factors, including oxygen and nutrient availability. The development of the early embryo before implantation *in vivo* requires minimal nutrients that are mainly derived from the oviduct fluid. One of these nutrients is pyruvate, which is the preferred energy substrate for 1-cell zygotes and essential for the development beyond the 2-cell stage and ZGA (see Figure 1A). Depletion of pyruvate in 1-cell mouse embryos leads to blockage in the 2-cell stage, highlighting the essential role of pyruvate in providing enough energy for the embryo during these first days of development (Brinster, 1965; Brown and Whittingham, 1991; Nagaraj et al., 2017). In line with this and reinforcing the importance of pyruvate metabolism in early development, genetic ablation of the subunit alpha of the pyruvate dehydrogenase (PDH) complex in oocytes leads to embryonic arrest as early as in the zygote stage, likely due to impaired mitochondrial metabolism (Johnson et al., 2007). Additionally, depletion of the mitochondrial pyruvate carrier in mice results in embryo developmental delay causing midgestation lethality (Bowman et al., 2016).

Early cleavage stage embryos show a very low oxygen consumption compared to the blastocyst stage (Houghton et al., 1996; Leese, 2012). However, the one-cell embryo utilizes OxPhos over glycolysis by fueling the TCA with pyruvate and relies on the respiration of the abundant number of maternally inherited mitochondria (Figures 1A,B) (Brinster, 1965). Nevertheless, the inherited mitochondria appear rounded and small at the 1- to 2-cell stages, whereas they become fully formed at the blastocyst stage (Brinster, 1967; Van Blerkom, 2009). Interestingly, pyruvate seems to be essential for the localization of certain TCA cycle-related enzymes into the nucleus in 2-cell stage embryos. Failure to re-localize these enzymes into the nucleus leads to epigenetic changes, such as decrease in specific acetylation and methylation histone marks (Nagaraj et al., 2017). The reduction in histone acetylation could be explained by the absence of pyruvate and, therefore, acetyl coenzyme A (acetyl-CoA) production in general; or by the failure of acetyl-CoA nuclear production due to absence of PDH nuclear localization. These exciting findings open the door for the plausible existence of a TCA cycle nuclear subnetwork that might be important for epigenetic modifications during early embryogenesis, and it is discussed in the next section.

Embryos grown in the absence of glucose become arrested in the compacted 8-cell stage, therefore glucose is indispensable for the transition from morula to blastocyst (Brown and Whittingham, 1991; Martin and Leese, 1995; Chi et al., 2020). This stage is characterized by a bivalent metabolism, where the embryo uses both glycolysis and TCA coupled to OxPhos to produce ATP (Folmes et al., 2012). Single-cell RNA transcriptomic analyses of human and mouse pre-implantation embryos (from zygote to blastocyst) indicate an upregulation of mitochondrial-related transcripts and genes involved in OxPhos around the morula to blastocyst transition (Xue et al., 2013; Yan et al., 2013). Accordingly, the blastocyst stage coincides with the highest peak of oxygen consumption in pre-implantation development (Figure 1B) (Houghton et al., 1996; Leese, 2012; Muller et al., 2019). Although not extensively studied, metabolic differences between the individual lineages that form the blastocyst also exist. Gene expression analysis and metabolic assessment of the bovine ICM and TE, demonstrate that the TE has increased metabolic demands in terms of lipid biogenesis and amino acids (AA) turnover compared to the ICM (Gopichandran and Leese, 2003; Ozawa et al., 2012). In addition, the TE of the mouse blastocyst shows a higher ratio of OxPhos compared to the ICM. The TE seems to consume significantly more oxygen, contain a higher number of mitochondria and produce more ATP than the ICM (Houghton, 2006). The fact that the TE shows different metabolic demands than the ICM might be explained by the activity of Na^+^, K^+^-ATPase pumps in the TE, which consume approximately 60% of the generated ATP to form the blastocoel (Houghton et al., 2003).

Recent metabolic analysis by Sharpley et al. (2021), has demonstrated that the abundance of glycolytic intermediates increases from the 2-cell stage to blastocysts, which is in accordance with the concomitant upregulation of glycolytic genes. In addition, this study also shows that glucose contribution to the TCA cycle metabolites, AA and acetyl-CoA is minor during preimplantation embryonic development. Instead, glucose is being partially used in the blastocyst stage, for nucleotide biosynthesis. Furthermore, flux analysis also included in this study suggests that pyruvate and lactate are the major contributors of fueling the TCA cycle from the 2-cell stage to blastocyst. Specifically, pyruvate and lactate contribute to acetyl-CoA synthesis and half of the TCA metabolites (citrate, isocitrate and αKG) (Sharpley et al., 2021). Interestingly, these three metabolites are synthesized by enzymes located in the nucleus of 2-cell embryos suggesting a potential role of nuclear TCA cycle in regulating the epigenome (Nagaraj et al., 2017).

The metabolic flexibility of the embryo increases as development continues. While the 1-/2-cell stage embryos are strictly dependent on the presence of lactate and pyruvate, more nutrients can sustain the development of later stages. ZGA and, therefore, the transcription of multiple metabolic genes combined with the activation of other pathways, allows the embryo to adapt to nutrient conditions and to have flexibility in the choice of energy substrate. Thus, beyond the 2-cell stage the embryo has preferred metabolic pathways, but little obligatory or essential pathways (Martin and Leese, 1995; Folmes et al., 2012; Sharpley et al., 2021). Upon implantation, the embryo is exposed to a highly oxygen-deprived environment that likely promotes a switch from aerobic to anaerobic metabolism. Glucose consumption seems to gradually rise from the morula stage, accelerates in the blastocyst stage and further increases upon embryo implantation (Johnson et al., 2003; Folmes et al., 2012). The majority of the glucose is converted into lactate in the first days of embryo implantation into the uterine wall (Figure 1B) (Johnson et al., 2003). The importance of a functional glycolysis upon embryo implantation is highlighted by the fact that mutations in glycolytic genes, such as *Gpi*, lead to early post implantation lethality (Merkle and Pretsch, 1992). In line with the above, recent single-cell embryo transcriptomic analyses across six mammalian species, suggest a potential inter-species conserved metabolic switch from a bivalent respiration towards a glycolytic metabolism during early embryonic development. Specifically, this metabolic switch might occur around the time of embryonic disk formation (Malkowska et al., 2022). The *in vitro* models for mouse pre- and post-implantation epiblast, mESC and mEpiSCs respectively, might recapitulate the metabolic differences observed in their *in vivo* counterparts. Similarly to the pre-implantation blastocyst, *in vitro* mESCs consume high levels of oxygen and are metabolically bivalent relying on both glycolysis and OxPhos for their energy production (Houghton et al., 1996; Leese, 2012; Zhou et al., 2012; Teslaa and Teitell, 2015). Additionally, mEpiSCs produce high levels of lactate, an indication of high glycolysis activity, also in line with mouse post-implantation embryos (Johnson et al., 2003; Zhou et al., 2012). However, single-cell transcriptomic analysis of established mESC lines from the ICM of a blastocyst, revealed that mESCs may acquire unique metabolic features that allow them to self-renew in a dish (Tang et al., 2010). Therefore, the switch from a normal developmental program to an infinite self-renewal in mESCs, might prevent them from acquiring the same metabolic state as the ICM *in vivo.* Nevertheless, the knowledge regarding metabolism of mouse embryos is currently limited, and stem cell models remain crucial to delineate new hypothesis, which subsequently should be tested in the embryo. Metabolomic studies and flux analysis of embryos in parallel with the *in vitro* stem cell models are required in order to assess the suitability of the current stem cell models.

### 3.2 Hypoxic environment upon embryo implantation leads to metabolic changes

Embryo implantation is associated with a drastic decrease in oxygen levels, from approximately 8% in the oviduct to 2% oxygen in the intrauterine tissue of several mammal species, including humans (Fischer and Bavister, 1993; Ottosen et al., 2006). This is accompanied by an inactivation of mitochondrial respiration in the implanted embryo (Brinster, 1965; Leese, 2012; Zhang et al., 2018). Indeed, post-implantation embryos seem to show an increased expression of lactate dehydrogenase A (LDHA), which favors the conversion of pyruvate to lactate and indicates a high glycolytic activity (Auerbach and Brinster, 1967; Johnson et al., 2003). Low oxygen tension leads to activation of a family of TFs called hypoxia inducible factors (HIFs) that regulate the expression of hundreds of genes involved in apoptosis, proliferation, angiogenesis, self-renewal and, as well, metabolism (Carmeliet et al., 1998; Ramírez-Bergeron et al., 2006; Goda and Kanai, 2012; Petruzzelli et al., 2014). HIFs are crucial for embryo development, since both *Hif1a* ^−/−^ and *Hif1b* ^−/−^ mice are embryonic lethal (Tian et al., 1997; Iyer et al., 1998; Kotch et al., 1999). HIF1 increases glucose intake and glycolysis by inducing the expression of glucose transporters and glycolytic enzymes (Semenza et al., 1994; Gleadle and Ratcliffe, 1997; Maxwell et al., 1997; Mathupala et al., 2001). In addition, HIF1 promotes the conversion of pyruvate to lactate by increasing the expression of LDHA (Semenza et al., 1996). HIF1 also decreases the mitochondrial respiration by inhibiting mitochondria biogenesis and activating mitophagy (Zhang et al., 2008, 2007). Altogether, the increased expression of HIF1 upon embryo implantation seems to be involved in rewiring the metabolic state of the embryo by reducing the activity of the TCA cycle and diverting the flux of pyruvate towards lactate synthesis. However, recent studies suggest that HIF1 might also be metabolically regulated. For example, αKG is a cofactor of prolyl hydroxylases (PHDs), which are necessary for HIF degradation (Koivunen et al., 2007; Durán et al., 2013). On the contrary, pyruvate and the TCA cycle intermediates succinate and fumarate were found to inhibit PHDs and, hence, stabilize and increase the levels of HIF TFs (Selak et al., 2005; Virtue and Vidal-Puig, 2011; Wang et al., 2019). Future studies could address if HIF expression might be controlled by the metabolic state of the cells depending on the context and the tissue. The crosstalk between the hypoxic environment and the metabolic state of cells is a yet unexplored field that has probable consequences for the chromatin state of the embryo. Elucidating the mechanistic basis of this relationship is necessary to better understand how the environment and metabolism shape the embryo development and adaptation to the maternal uterus. The hypoxic environment is a common feature of the early developing embryo and several cancers. Therefore, common lessons might be learned by comparing these two systems.

### 3.3 Onco- and stem-cell metabolism: The possible role of 2-hydroxyglutarate (2-HG) in development

Embryo implantation is accompanied by an increased glucose uptake and lactate synthesis, and thereby probably enhanced glycolysis, as described in the previous section (Johnson et al., 2003; Folmes et al., 2012). In the same line, *in vitro* mESCs switching to primed mEpiSCs increase the number of glucose transporters and the expression of glycolytic genes (Zhou et al., 2012; Kim et al., 2015; Yu et al., 2019). The metabolic profile of some cancer cells resembles those seen in specific stages of embryonic development. The tumor microenvironment is very heterogeneous, hence cancer cells have to adapt to various levels of nutrients and oxygen availability in order to survive and proliferate (Ward and Thompson, 2012; Yoshida, 2015). Therefore, tumor cells show a high level of plasticity and metabolic reprogramming similarly to the embryo. Several types of cancer cells rewire their metabolism towards aerobic glycolysis and increased lactate production, even at the presence of sufficient oxygen to support OxPhos, in a process known as the Warburg effect (Warburg, 1956; Eales et al., 2016). The increase of the glycolytic program and glucose uptake in tumor cells is achieved by the overexpression of key glycolytic enzymes and glucose transporters, such as GLUT1 (Altenberg and Greulich, 2004). Additionally, many tumors overexpress the enzyme pyruvate dehydrogenase kinase (PDK), which inhibits PDH, resulting in decreased conversion of pyruvate towards acetyl-CoA (Hur et al., 2013; Sutendra and Michelakis, 2013; Schell et al., 2014). This metabolic rewiring towards a more glycolytic metabolism is a feature shared by cancer and mEpiSCs (Varum et al., 2011; Rodrigues et al., 2015).

Of relevance, the oncometabolite 2-HG was proposed to play an unexpected role in early embryogenesis. 2-HG is a structurally similar metabolite to αKG that can be generated by reducing the ketone group of αKG to a hydroxyl group. In connection to epigenetics, 2-HG is a competitive inhibitor of αKG-dependent dioxygenases, thus indirectly regulates the levels of DNA and histone methylation, among others (Figure 2) (Xu et al., 2011). To-date, 2-HG has been mainly considered an oncometabolite promiscuously synthesized by mutant isocitrate dehydrogenase 1/2 (*Idh1/2*) enzymes, which are altered in 70–80% of grade II/III gliomas and glioblastomas among other cancers (Dang et al., 2009; Gagné et al., 2017; Medeiros et al., 2017; Yan et al., 2009). In addition, it was recently reported that promiscuous activity of LDH and malate dehydrogenase (MDH) can also produce 2-HG under certain conditions (Intlekofer et al., 2017). In the embryonic development context, Sharpley et al. (2021), reported high levels of 2-HG in 2-cell stage embryos that decreased in morula and blastocyst stages. Interestingly, the levels of αKG and 2-HG were proportionally inverse. In agreement, a recent metabolomics study reported that indeed 2-cell embryos have increased levels of 2-HG compared to blastocysts. Specifically, the enantiomer L-2-HG previously found to be produced under hypoxic and acidic conditions, was found to be highly enriched in MII oocytes, zygote and 2-cell stage embryos, steadily decreasing during embryonic development reaching the lowest levels at the blastocyst stage (Intlekofer et al., 2017, 2015; Zhao et al., 2021). Treatment of developing embryos from the zygote to 4-cell stage with permeable L-2-HG led to epigenetic abnormalities, developmental delay and morphological aberrations. These phenotypes were partially rescued when treating the embryos with permeable αKG (dm-αKG), indicating that the ratio αKG/2-HG might be important for these stages of embryogenesis (Zhao et al., 2021).

The above-mentioned examples highlight the high degree of overlap that exists in the context of metabolism between cancer and stem cells. For therapeutic reasons, the metabolic profile of cancer cells has been extensively studied when compared to stem cells. Future investigations should address whether lessons learned from cancer cells can help us to decipher how metabolism influences cellular identity in the early embryo. Furthermore, various types of tumors with poor prognosis harbor a population of uncharacterized cancer stem cells; hence, knowledge on embryonic stem cells could provide information for the development of potential therapies against cancer stem cells.

### 3.4 Signaling events regulate the levels of epi-metabolites during development

Metabolic changes can directly influence the activity of signaling pathways and *vice versa*. One question here is: do the main signaling pathways, which drive early embryonic development, also control the levels of epi-metabolites? STAT3 is a known TF that directly regulates the expression of naïve pluripotent genes and cellular metabolism by promoting OxPhos (Niwa et al., 1998; Bourillot et al., 2009; Niwa et al., 2009; Wegrzyn et al., 2009; Tai and Ying, 2013; Carbognin et al., 2016). Recently, a new role for LIF-STAT3 signaling has been proposed. Mitochondrial STAT3 directly regulates the levels of mitochondrial αKG, which in turns promotes nuclear genome hypomethylation, characteristic of naïve pluripotent stem cells, *via* downregulation of *de novo* DNA methylases DNMT3A/B (Betto et al., 2021). Accordingly, hESCs treated with dm-αKG also show reduced expression of *DNMT3B*, although this study points to a more bidirectional link between DNMT3B and αKG, since *DNMT3B*-null cells exhibit high levels of αKG (Cieslar-Pobuda et al., 2020). In addition, pre-implantation *Stat3* ^*−/−*^ mouse embryos show upregulation of post-implantation markers, such as *Otx2* and *Dnmt3a/b,* and die soon after implantation (Takeda et al., 1997; Betto et al., 2021). To evaluate whether the metabolic control of DNA methylation is taking place *in vivo*, the levels of αKG in *Stat3* ^*−/−*^ blastocysts should be measured.

The Hippo and the mammalian target of rapamycin (mTOR) are important signaling pathways for embryonic development and closely linked to metabolism. The Hippo-YAP/TAZ signaling pathway is required for proper TE lineage specification and is conserved in both mouse and human embryos (Kono et al., 2014; Hirate et al., 2015; Gerri et al., 2020; Ibar and Irvine, 2020; Meinhardt et al., 2020). The inhibition of Hippo signaling leads to nuclear translocation of YAP/TAZ and upregulation of their target genes (Kwon et al., 2021). Glucose metabolized by the hexosamine biosynthesis pathway (HBP) leads to glycosylation and subsequent nuclear localization of YAP promoting TE cell fate (Chi et al., 2020). In addition, the mTOR pathway, which can be activated by glucose-dependent nucleotide synthesis by the pentose phosphate pathway (PPP), leads to translation of TFAP2C (Chi et al., 2020). The complex YAP-TFAP2C-TEAD4 is critical for TE specification and controls the expression of TE-specific markers such as GATA3 and CDX2 (Strumpf et al., 2005; Yagi et al., 2007; Ralston et al., 2010; Cao et al., 2015; Gerri et al., 2020). Glucose-deprivation or inhibition of the HBP or PPP resulted in loss of CDX2 expression, whereas NANOG and OCT4 were unaffected (Chi et al., 2020). This constitutes an exciting example of how metabolism directly influences the priming of specific cellular lineages as early as the morula stage. Future studies are necessary in order to delineate the extent of lineage-specific TF regulation by glycosylation during cell fate decisions in early embryogenesis.

Metabolism is regulated by the activity of signaling pathways as described above. However, cellular signaling is also the sensor of metabolic changes. For instance, changes in the nutrient availability or bioenergetics resulting in decreased intracellular ATP lead to mTOR inactivation [reviewed in (Kim et al., 2013)]. Another example occurs in intestinal stem cells, where high levels of OxPhos leads to increased generation of reactive oxygen species (ROS), which can activate the MAPK pathway (Rodríguez-Colman et al., 2017). The role of metabolism regulating key signaling pathways during early embryogenesis remains poorly understood.

## 4 The interplay between metabolism and epigenetics shapes stem cell identity

Intracellular metabolite levels are determined by the activity of intrinsic metabolic pathways and extrinsic cues from the environment, such as nutrient availability and growth factor signaling (Schvartzman et al., 2018). As previously mentioned, several of the metabolites, act as substrates, co-factors or inhibitors to modulate the activity of histone and DNA modifying enzymes (Katada et al., 2012; Lu and Thompson, 2012). The mechanisms of action by which metabolism regulates epigenetics were already extensively reviewed elsewhere (Ryall et al., 2015; Harvey et al., 2016; Harvey et al., 2019). Here we focus on the role of epi-metabolites, specifically the TCA intermediate αKG, in regulating chromatin states and cell fate transitions.

### 4.1 The role of the TCA cycle in regulating epigenetics and cell fate

Changes on the TCA metabolite levels affect the activity of αKG-dependent dioxygenases, which ultimately regulate pluripotency in early stages of development (Nottke et al., 2009; Shen et al., 2017; Gökbuget and Blelloch, 2019; Jambhekar et al., 2019). Of importance, αKG is the main co-activator and substrate of the family of αKG-dependent dioxygenases, which includes Jumonji C-domain lysine demethylases (JmjC-KDMs), ten-eleven translocation (TETs) methylcytosine dioxygenases and PHDs. All these enzymes consume αKG and oxygen and produce succinate and CO_2_. On the contrary, 2-HG and the TCA metabolites succinate and fumarate can function as competitive inhibitors of αKG-dependent dioxygenases (see Figure 2) (Reid et al., 2017).

During recent years, the link between metabolism and cellular identity has become apparent and a rising number of studies are challenging the idea of metabolism being merely a passive player in regulating cell fate and embryogenesis. For instance, *in vitro*, αKG and OxPhos regulate pluripotency and differentiation (Tischler et al., 2019). Indeed, Tischler et al. (2019), showed that cells undergoing mESC-to-mEpiLC transition treated with the glycolysis inhibitor 2-deoxy-D-glucose (2-DG) or with permeable dm-αKG, are prevented from exiting naïve pluripotency. The same investigation showed that culturing mESCs with dm-αKG in the absence of 2i is sufficient to preserve the naïve state (Tischler et al., 2019). In line with this study, naïve mESCs cultured in 2i/L have a substantial higher ratio of αKG/succinate compared to mESCs cultured in S/L (Carey et al., 2015). Both studies suggest that the high levels of αKG in naïve mESCs support the activity of JmjC-KDMs and TETs in maintaining the naïve cellular identity by preserving a hypomethylated chromatin landscape and DNA. In fact, modifying the levels of αKG/succinate leads to changes in histone methylation status of several histone marks, such as H3K9 and H3K27(Carey et al., 2015). In addition to promoting self-renewal of naïve mESCs, αKG accelerates differentiation of primed human pluripotent stem cells (hPSCs), whereas succinate or αKG depletion delay their differentiation. Although in this case αKG seems to promote differentiation and not self-renewal, the mechanism behind is consistent with the one described by Carey et al. (2015). Treatment of primed H9 hPSCs after 6 days of neuroectoderm differentiation with dm-αKG leads to increased DNA hydroxymethylation and decreased specific lysine methylation marks (TeSlaa et al., 2016). Another example of αKG enhancing differentiation is the role of αKG in promoting primordial germ cell (PGC) fate. Specifically, αKG preserves the epigenetic landscape of PGC-competent mEpiLCs, hence stabilizing their transient developmental PGC potential (Tischler et al., 2019). However, how specific is the effect of treating cells with exogenous dm-αKG? Could the observed phenotypes be partially due to an off-target effect of exogenous dm-αKG or could dm-αKG be metabolized to 2-HG and/or succinate and affect the activity of histone and DNA modifying enzymes? The above-mentioned studies use non-toxic concentrations of dm-αKG, however a recent paper questions the use of dm-αKG, and in general cell-permeable esterified analogs, due to their potential independent effects on cellular metabolism, including extracellular acidification or inhibitory effects on glycolysis and OxPhos (Parker et al., 2021). Therefore, future studies should corroborate the above-mentioned findings by finely tuning the intracellular levels of αKG.

These results demonstrate a link between TCA metabolism and epigenetic control of cell state transitions in early embryonic development and open the door for further exploration of this complex crosstalk in other cell fate decisions and lineage commitment. In this regard, one might wish to assess the role of metabolism during the first lineage commitment in the morula, where blastomeres become either part of the TE and ICM, two metabolically very distinct cellular lineages. Additionally, the ICM of the blastocyst and the post-implanted EPI might show highly different metabolic profiles. Therefore, the study of how metabolism influences cell fate and cell commitment at peri-implantation is crucial for understanding the process of a successful embryo implantation, the moment where most human pregnancies fail (Rinehart, 2007).

### 4.2 Acetyl-CoA and one-carbon metabolism impact pluripotency through epigenetic regulation

S-adenosylmethionine (SAM) is the cell’s principal methyl donor and is generated as an intermediate of the methionine and folate cycles, which are part of the one-carbon metabolism (Clare et al., 2019). SAM is the substrate for histone and DNA methyltransferases, (i.e. HMTs and DNMTs) (Figure 2). Controlling the intracellular levels of SAM is crucial to maintain undifferentiated hESCs and to regulate their differentiation. Depletion of SAM leads to reduced H3K4me3 levels and subsequent increased differentiation in hESCs and mESCs (Shyh-Chang et al., 2013; Shiraki et al., 2014). Due to its important role, specific systems evolved to fine-tune the intracellular levels of SAM. For example, naïve hESCs show a high expression of the enzyme N-methyltransferase (NNMT), which consumes SAM and, hence is not available for histone methylation (Sperber et al., 2015). This is of special relevance during the transition from naïve to primed pluripotency, where the levels of NNMT decrease and hence the levels of SAM increase. In line with this, NNMT-KO naïve hESCs show upregulation of SAM, H3K27me3, H3K9me3 and in general a gene expression profile shifted towards the primed state (Sperber et al., 2015). Taken together, a functional methionine metabolism is crucial for regulating cell states through epigenetic networks. Future studies should address the importance of regulating the SAM levels and the impact on the epigenetic state during early embryonic development.

Acetyl-CoA is a metabolic intermediate synthesized by a number of reactions, such as glycolysis, catabolism of some AA and oxidation of fatty acids. In addition, acetyl-CoA is closely linked to epigenetics, since it constitutes the substrate of histone acetyltransferases (HATs) (Figure 2). Cytoplasmic TCA-derived acetyl-CoA production by ATP-citrate lyase (ACLY) is upregulated in mESCs cultured in S/L and primed hESCs resulting in increased acetylation of H3K9 and H3K27, hence maintaining an opened chromatin state (Moussaieff et al., 2015). However, inhibition of glycolysis or ACLY and therefore decreased levels of acetyl-CoA led to premature differentiation of mESCs and hESCs (Moussaieff et al., 2015). Conversely, a recent study suggests that ACLY is required for exiting naïve pluripotency of mESCs. Inhibition of ACLY leads to expression of naïve markers even in the absence of 2i/LIF (Arnold et al., 2022). All of these results show a link between metabolism, chromatin states and maintaining or exiting pluripotency depending on the cell state. The regulation of acetyl-CoA levels appears to be crucial for proper mouse embryonic development. In this line, homozygous *Acly-*KO mice, hence with decreased cytoplasmic acetyl-CoA levels, are embryonic lethal around E8.5 (Beigneux et al., 2004). The importance of acetyl-CoA in early stages of embryonic development and the possible link with epigenetic regulation in the embryo remains unknown.

### 4.3 Compartmentalized metabolism, chromatin landscapes and cell fate decisions

Cellular metabolism takes place in different organelles. The most prominent is mitochondrial metabolism, which is mainly associated with energy production and generation of building blocks for macromolecules synthesis. However, metabolic reactions also occur in other compartments. A non-canonical TCA (ncTCA) cycle has been described in mESCs. As previously shown, citrate can be exported from the mitochondria to the cytosol and be converted to acetyl-CoA and oxaloacetate (OAA) by ACLY, a mechanism that is found upregulated in some types of cancer cells (see Figure 2) (Hatzivassiliou et al., 2005). The acetyl-CoA generated in the cytoplasm is used for protein acetylation and lipid biogenesis, while the OAA is converted to malate and re-directed to the mitochondria to complete the ncTCA (Arnold et al., 2022). Interestingly, mESC exiting naïve pluripotency rely more on the ncTCA to survive and to generate TCA-cycle intermediates, as demonstrated by flux analysis where the concentration of unlabeled citrate, upon addition of labelled glucose, progressively increased after 2i removal (Arnold et al., 2022). The differential activity of the ncTCA depending on the environment where mESCs are grown highlights the importance of metabolism outside the mitochondria. Future studies should address the link between ncTCA and histone acetylation, since the cytoplasmic acetyl-CoA synthesis has been linked to histone acetylation in embryonic stem cells (Moussaieff et al., 2015). Of special attention for our review is the role of nuclear metabolism and its implications on cell fate and embryonic development, and it is discussed in the following section.

#### 4.3.1 What is the role of nuclear localization of metabolic enzymes?

Recent discoveries revealed that some TCA-cycle enzymes are located in the nucleus of several cell types, including mESCs and mouse embryos, as early as in the 2-cell stage (Yogev et al., 2010; Sutendra et al., 2014; Jiang et al., 2015; Nagaraj et al., 2017; Wang et al., 2017; Chen et al., 2018; Liu et al., 2021; Kafkia et al., 2022). Furthermore, some studies described a possible TCA cycle subnetwork that takes place within the nucleus. Nagaraj et al. (2017) demonstrated the existence of a nuclear pyruvate-to-αKG route involving pyruvate carboxylase and dehydrogenase, and as well a nuclear localization of the first half of the TCA cycle enzymes, the so-called class I enzymes. The presence of these enzymes in the nucleus provides the embryo with acetyl-CoA and αKG, which are epi-metabolites used for either direct acetylation of histone tails or activation of histone and DNA demethylases, respectively (Figure 2). Indeed, pyruvate-depleted 2-cell embryos show a drastic reduction of specific histone acetylation and methylation marks and an arrest in development (Nagaraj et al., 2017). Importantly, this 2-cell block is rescued by supplementation of αKG, reinforcing the role of αKG in regulating chromatin states and development. However, localization of class I TCA cycle enzymes was not detected beyond the 2-cell stage in this study, suggesting a more restricted role in the very early stages of embryo development, such when ZGA takes place. Future studies should corroborate these findings during embryonic development of other species and delve more into the specific function of nuclear metabolic enzymes.

Re-localization of TCA cycle enzymes in the nucleus has been further demonstrated and explored in other mammalian model systems and under different contexts. For example, the presence of aconitase 2 (ACO2), IDH1, the subunit G of the IDH3 complex (IDH3G), oxoglutarate dehydrogenase (OGDH) subunit from the oxoglutarate dehydrogenase complex (OGDC) in the nucleus has been described using the human cervical cancer cells HeLa (Kafkia et al., 2022). In addition, all the above-mentioned enzymes as well as citrate synthase (CS) succinyl-CoA synthetase (SCS), fumarate hydratase (FH) and MDH2 presence in the nucleus of mouse liver cells, human hepatoma HepG2 cells and human breast adenocarcinoma MCF7 cells has also been reported (Liu et al., 2021). Finally, nuclei of mESCs have also a strong presence of ACO2 and OGDH (see Figure 2) (Kafkia et al., 2022). Taken together, the re-localization of metabolic enzymes into the nucleus seems to be linked with the presence of a functional TCA cycle nuclear subnetwork. A question arises here: is there a nuclear production of epi-metabolites or do they diffuse into the nucleus from the cytoplasm? Metabolites can freely diffuse from the cytoplasm into the nucleus due to the large size of the nuclear pores (Wente and Rout, 2010). However, metabolic flux analysis of isolated nuclei from HeLa cells revealed the existence of citrate-to-succinate, glutamine-to-fumarate and glutamine-to-aspartate nuclear pathways (Kafkia et al., 2022). This suggests the presence of a nuclear TCA cycle subnetwork rather than a free metabolite diffusion scenario (Kafkia et al., 2022). The presence of this nuclear subnetwork, especially in embryos and in mESC, sets the grounds for the study of how nuclear metabolic reprogramming regulates chromatin and gene expression. Metabolomic flux analysis restricted in the nucleus of mESCs would improve our understanding of the most prominent and important pathways of nuclear metabolism in different stages of embryonic development. However, no such technology has been developed yet. Future studies should also address the presence of nuclear TCA enzymes beyond the 2-cell stage and the precise role in regulating embryo development. Importantly, nuclear presence of PDH was also reported in human embryos, although at a slightly later stage of embryo development (Nagaraj et al., 2017). Interestingly, this stage of human development coincides with the ZGA of human embryos, the same stage where metabolic enzymes are re-localized to the nucleus in mouse embryos. Thus, this re-localization might be linked to ZGA of embryos and constitute a conserved mechanism among species.

The plausible existence of a nuclear TCA cycle subnetwork opens the door to study the role of metabolic enzymes within the nucleus. One attractive hypothesis is that chromatin location of metabolic enzymes allows for locus-specific epi-metabolite production and/or consumption. For example, OGDH directly binds the histone acetyltransferase KAT2A and generates succinyl-CoA, which is used by KAT2A to succinylate H3K79, and enhance transcription and tumor proliferation in glioma and HEK293 cell lines (Figure 2) (Wang et al., 2017). Another TCA cycle enzyme with a described nuclear role is the chromatin-localized FH. As previously mentioned, fumarate is an inhibitor of αKG-dependent dioxygenases (Xiao et al., 2012). Local production of nuclear fumarate leads to inhibition of KDM2B and subsequent increased H3K36 dimethylation, which in turn results in enhanced cell survival by promoting DNA repair at double strand breaks (Jiang et al., 2015). Similarly, nuclear production of acetyl-CoA by acetyl-CoA synthetase 2 (ACCS2) at neuron-specific genes promotes histone acetylation in neurons undergoing differentiation. Loss of ACCS2 leads to decreased histone acetylation affecting long-term memory and gene expression (Mews et al., 2017). Furthermore, the global levels of histone acetylation in cancer cells are also dependent on nuclear acetyl-CoA production by ACLY (Wellen et al., 2009). In addition, local production of NAD^+^ by chromatin-bound NMNAT1 activates the histone deacetylase SIRT1, resulting in transcriptional regulation of specific genes in breast cancer cells MCF-7 (Zhang et al., 2009). All these studies suggest an exciting view of metabolism, which locally generates microenvironments of epi-metabolites that are used by chromatin modifying enzymes. Hence, this scenario points to the role of metabolic enzymes directly regulating the transcription of genes. The expression of genes encoding for metabolic enzymes varies among different stages of development, therefore it is not far-off to think about a scenario where metabolic enzymes might be directly regulating the expression of specific developmental genes during embryogenesis.

The nuclear production of epi-metabolites begs the question: how are metabolic enzymes being imported into the nucleus? One mechanism that could explain this is the presence of a nuclear localization signal (NLS). This type of sequence has been identified in the DLST subunit of the OGDC complex (Wang et al., 2017). Moreover, by the use of the ELM database, Kafkia et al. (2022), predicted the presence of a NLS in almost all the TCA class I enzymes (Nagaraj et al., 2017). Transportation of enzymes into the nucleus by mitochondrial-derived vesicles (MDVs) or nuclear import by recognition of *O*-glycosylation residues, have also been proposed as a plausible mechanism (Szollosi and Szollosi, 1988; Sugiura et al., 2014; Nagaraj et al., 2017). Additionally, post-translational modifications (PTMs) of enzymes constitute an important mechanism for protein translocation from cytoplasm into the nucleus. Indeed, phosphorylation of pyruvate kinase isoform 2 (PKM2) leads to its nuclear translocation and subsequent function as co-activator of b-catenin-target genes (Vergara et al., 2018). Therefore, analysis of plausible and relevant PTMs in metabolic enzymes might help to elucidate the mechanism of nuclear localization of those enzymes. Interestingly, several metabolic enzymes show low-complexity regions and form molecular condensates under certain conditions (Prouteau and Loewith, 2018). This might suggest that metabolic enzymes could undergo phase separation and provide a nuclear metabolome in certain metabolic and cellular contexts. Finally, histone reservoirs for metabolites might exist. For example, certain chromatin modifiers can establish methylation and acetylation reservoirs in histones that are ready to be rapidly mobilized to sustain cellular metabolism in certain contexts (Kurdistani, 2014; Ye et al., 2017; Ye and Tu, 2018). Recently, it has been suggested that the high presence of methyl or acetyl groups in histones not always correlates with the locus transcriptional activity (Ye and Tu, 2018). Therefore, these metabolite histone reservoirs might be used for regulating the epigenetic and metabolic state of cells in certain contexts. In conclusion, the mechanism of translocation of metabolic enzymes into the nucleus remains an open question. Of special interest, would be to study the import mechanisms in the context of embryo development, since depletion of pyruvate in the 2-cell stage leads to failure of nuclear localization of metabolic enzymes and embryonic arrest (Nagaraj et al., 2017).

## 5 Concluding remarks

The novel view of metabolism goes beyond generating biomass and energy for cellular and embryonic growth. In this review, we have outlined the emerging non-canonical role of metabolism in regulating epigenetics and cell fate, especially in the context of embryonic development. Although, the above-described findings are exciting and establish the grounds for future research, the field of epi-metabolomics faces many challenges that should be addressed. One of the most difficult issues to tackle is how the specificity is achieved in regard to metabolic regulation of chromatin states. How do certain metabolic enzymes regulate specific chromatin marks that mediate transcriptional responses? Are there specific chromatin marks being regulated by the activity of a unique metabolic enzyme? How sensitive are those chromatin-modifying enzymes to metabolite fluctuations? Furthermore, the study of novel histone marks, such as lactylation, which might be highly sensitive to metabolic changes, could be of significance in the context of gene regulation during early embryonic development. Preliminary studies suggest that lactate, especially under certain physiological conditions, such as hypoxia, can also exist on the form of lactyl-CoA (Zhang et al., 2019; Snyder et al., 2020). However, the mechanisms of action involving histone lactylation and gene expression, and the enzymes responsible for lactyl-CoA synthesis and the writers, erasers and readers of histone lactylation remain unknown (Figure 2). In this same context, the existence of other acylation histone PTMs, including succinylation, malonylation, glutarylation, butyrylation or propionylation among others, contribute to chromatin compaction and likely to gene expression [reviewed in (Nitsch et al., 2021)]. For instance, histone propionylation and butyrylation, most often located in promoters together with acetylation, correlate with active transcription in several *in vitro* cell lines and mouse spermatogenic cells, respectively (Goudarzi et al., 2016; Kebede et al., 2017). The impact of these acyl-CoA metabolites in the complexity of gene expression regulation and cell fate decisions during early embryogenesis remains unknown and constitutes an interesting matter of future studies.

The majority of studies performed to-date are based on *in vitro* stem cell models or transcriptomic analysis from embryos. Currently, stem-cell based embryo models are being used in order to recapitulate snapshots of mouse and human embryo development (Sozen et al., 2021, 2019; Liu et al., 2021; Yu et al., 2021; Kagawa et al., 2022). Understanding the role of chromatin as a sensor of metabolic states will help us elucidate the importance of metabolism as another layer of gene regulation and the establishment of epigenetic memory during mammalian development. Many open questions remain in this respect. Specifically, to what extent metabolism influences epigenetic memory? How long does this memory persist? And what might be its physiological function? Addressing this promises to uncover new regulatory principles of human development and aid in the design of more efficient *in vitro* fertilization treatments.
